# Early Signs Monitoring to Prevent Relapse in Psychosis and Promote Well-Being, Engagement, and Recovery: Protocol for a Feasibility Cluster Randomized Controlled Trial Harnessing Mobile Phone Technology Blended With Peer Support

**DOI:** 10.2196/15058

**Published:** 2020-01-09

**Authors:** Andrew Gumley, Simon Bradstreet, John Ainsworth, Stephanie Allan, Mario Alvarez-Jimenez, Louise Beattie, Imogen Bell, Max Birchwood, Andrew Briggs, Sandra Bucci, Emily Castagnini, Andrea Clark, Sue M Cotton, Lidia Engel, Paul French, Reeva Lederman, Shon Lewis, Matthew Machin, Graeme MacLennan, Claire Matrunola, Hamish McLeod, Nicola McMeekin, Cathrine Mihalopoulos, Emma Morton, John Norrie, Frank Reilly, Matthias Schwannauer, Swaran P Singh, Lesley Smith, Suresh Sundram, David Thomson, Andrew Thompson, Helen Whitehill, Alison Wilson-Kay, Christopher Williams, Alison Yung, John Farhall, John Gleeson

**Affiliations:** 1 Glasgow Institute of Health and Wellbeing Glasgow Mental Health Research Facility University of Glasgow Glasgow United Kingdom; 2 NHS Greater Glasgow and Clyde Glasgow United Kingdom; 3 Division of Informatics, Imaging, and Data Sciences School of Health Sciences University of Manchester Manchester United Kingdom; 4 Orygen The National Centre of Excellence in Youth Mental Health Melbourne Australia; 5 Centre for Youth Mental Health University of Melbourne Melbourne Australia; 6 Division of Mental Health and Wellbeing University of Warwick Warwick United Kingdom; 7 London School of Hygiene and Tropical Medicine London United Kingdom; 8 Division of Psychology and Mental Health School of Health Sciences University of Manchester Manchester United Kingdom; 9 Greater Manchester Mental Health NHS Foundation Trust Manchester United Kingdom; 10 La Trobe University Melbourne Australia; 11 NorthWestern Mental Health Melbourne Australia; 12 NHS Research Scotland Mental Health Network Glasgow United Kingdom; 13 Deakin University Melbourne Australia; 14 Manchester Metropolitan University Manchester United Kingdom; 15 School of Computing and Information Systems Melbourne School of Engineering University of Melbourne Melbourne Australia; 16 The Centre for Healthcare Randomised Trials University of Aberdeen Aberdeen United Kingdom; 17 Glasgow Institute of Health and Wellbeing University of Glasgow Glasgow United Kingdom; 18 Australian Catholic University Melbourne Australia; 19 Usher Institute University of Edinburgh Edinburgh United Kingdom; 20 Scottish Recovery Network Glasgow United Kingdom; 21 School of Health and Social Sciences University of Edinburgh Edinburgh United Kingdom; 22 Monash University Melbourne Australia

**Keywords:** schizophrenia, psychosis, relapse, mHealth, randomized controlled trial

## Abstract

**Background:**

Relapse in schizophrenia is a major cause of distress and disability and is predicted by changes in symptoms such as anxiety, depression, and suspiciousness (early warning signs [EWSs]). These can be used as the basis for timely interventions to prevent relapse. However, there is considerable uncertainty regarding the implementation of EWS interventions.

**Objective:**

This study was designed to establish the feasibility of conducting a definitive cluster randomized controlled trial comparing Early signs Monitoring to Prevent relapse in psychosis and prOmote Well-being, Engagement, and Recovery (EMPOWER) against treatment as usual (TAU). Our primary outcomes are establishing parameters of feasibility, acceptability, usability, safety, and outcome signals of a digital health intervention as an adjunct to usual care that is deliverable in the UK National Health Service and Australian community mental health service (CMHS) settings. We will assess the feasibility of candidate primary outcomes, candidate secondary outcomes, and candidate mechanisms for a definitive trial.

**Methods:**

We will randomize CMHSs to EMPOWER or TAU. We aim to recruit up to 120 service user participants from 8 CMHSs and follow them for 12 months. Eligible service users will (1) be aged 16 years and above, (2) be in contact with local CMHSs, (3) have either been admitted to a psychiatric inpatient service or received crisis intervention at least once in the previous 2 years for a relapse, and (4) have an International Classification of Diseases-10 diagnosis of a schizophrenia-related disorder. Service users will also be invited to nominate a carer to participate. We will identify the feasibility of the main trial in terms of recruitment and retention to the study and the acceptability, usability, safety, and outcome signals of the EMPOWER intervention. EMPOWER is a mobile phone app that enables the monitoring of well-being and possible EWSs of relapse on a daily basis. An algorithm calculates changes in well-being based on participants’ own baseline to enable tailoring of well-being messaging and clinical triage of possible EWSs. Use of the app is blended with ongoing peer support.

**Results:**

Recruitment to the trial began September 2018, and follow-up of participants was completed in July 2019. Data collection is continuing. The database was locked in July 2019, followed by analysis and disclosing of group allocation.

**Conclusions:**

The knowledge gained from the study will inform the design of a definitive trial including finalizing the delivery of our digital health intervention, sample size estimation, methods to ensure successful identification, consent, randomization, and follow-up of participants, and the primary and secondary outcomes. The trial will also inform the final health economic model to be applied in the main trial.

**Trial Registration:**

International Standard Randomized Controlled Trial Number (ISRCTN): 99559262; http://isrctn.com/ISRCTN99559262

**International Registered Report Identifier (IRRID):**

DERR1-10.2196/15058

## Introduction

### Background

Relapse influences the long-term course of psychosis with rates identified as 28% (range 12%-47%), 43% (range 35%-54%), 54% (range 40%-63%) at 1, 1.5, 2, and 3 years follow-up, respectively [1], and more recent evidence shows similar relapse rates [2,3]. Relapse is also associated with poorer response to subsequent antipsychotic treatment [4]. Relapses can occur in up to 80% of participants at the 10-year follow-up [5] and can lead to higher inpatient and outpatient costs [6].

One important predictor of relapse is lack of acceptance of treatment and unplanned discontinuation of antipsychotic medication [1]. Poorer adherence often signals a lack of engagement with services and failure of services to build a collaborative working alliance [7]. Nonadherence to antipsychotic treatment is predicted by poorer insight, previous experience of involuntary treatment, poorer premorbid functioning, comorbid substance misuse, forensic history, poor relationship with the prescriber, greater deprivation, and transfer to secondary care [8-10]. Relapse itself is an important marker of the severity and complexity of illness and is predicted by previous suicide attempts [11]; depression, hostility, and embarrassment [12]; poorer premorbid functioning; family criticism; substance misuse; social isolation [1]; and negative interpersonal style (possibly linked to poorer utilization of social support) [13].

Birchwood et al [14] pioneered the development of systematic early signs monitoring for relapse and its integration into routine care. It is now known that relapse is the culmination of a process of changes that commence days and sometimes weeks before psychosis symptoms reemerge or are exacerbated. These early warning signs (EWSs) include affective changes and incipient psychosis. These EWSs can be detected as early as 8 weeks before rehospitalization [15]. A systematic review [16] found that the sensitivity of early signs to relapse (proportion of relapses correctly predicted) ranged from 10% to 80% (median 61%) and specificity (proportion of nonrelapses correctly identified) ranged from 38% to 100% (median 81%). Detection of relapse was improved by more frequent monitoring (at least fortnightly) and by the inclusion of both psychotic and affective symptoms.

A significant barrier to relapse prevention is fear of help seeking arising from previous experiences of relapse [17]. For example, people may avoid calling their care coordinator in the context of an increase in EWSs for fear of being admitted to the hospital. Research has demonstrated that fear of relapse is linked to more traumatic experiences of psychosis and hospital admission and greater fear of symptoms, such as voices and paranoia [18]. In a randomized controlled trial (RCT) of relapse detection, fear of relapse contributed independently to the prediction of relapse (sensitivity=72%, 95% CI 52-86; specificity=46%, 95% CI 32-60) compared with EWSs (sensitivity=79%, 95% CI 62-89; specificity=35%, 95% CI 23-50). Fear of recurrence was also associated with greater depression, feelings of entrapment, self-blame, and shame [19]. Therefore, it is important to include fear of recurrence in the monitoring of EWSs.

Fear of illness and stigma are closely related to emotional distress [20] and to poorer insight into schizophrenia [8]. Feelings of fear, depression, and helplessness are common emotional experiences before full relapse [21]. In an effort to minimize the stigma of illness and prevent relapse, people can adopt avoidant coping styles that are associated with increased risk of relapse [20]. These coping styles are associated with greater insecurity in relationships, lower self-esteem, lower levels of adherence, and reluctance to seek help in a crisis. Reluctance to seek help may result from greater fear of relapse arising from experiences of involuntary admission. In this sense, avoidance of help seeking can be understood from the perspective that people with experience of psychosis are attempting to minimize or avert the adverse consequences of help seeking based on their lived experience. A recent systematic review [22] found that greater difficulties in forming relationships were associated with poorer engagement with services, more problematic relationships with staff, and more frequent and longer hospital admissions. In sum, the detection of and action following EWSs may be constrained by poor relationships between service providers and people using services, avoidance of help seeking, perceived stigma, fear of relapse, and reluctance to disclose EWSs.

A Cochrane review focused on the effectiveness of interventions targeting recognition and management of EWSs of relapse in schizophrenia [23]. Significant effects in favor of EWS interventions were found for the number of participants relapsing (15 RCTs; n=1502; risk ratio [RR] 0.53, 95% CI 0.36-0.79) and the number of participants being rehospitalized (15 RCTs; n=1457; RR 0.48, 95% CI 0.35-0.66); however, the methodological quality of the trials was poor in terms of randomization, concealment, and blindness. Future EWS interventions need to address these methodological problems that limit their generalizability to usual care. Until this happens, EWS interventions cannot be recommended for routine implementation in health services [23].

An important aspect of service provision for those people at greatest risk of relapse is having access to an integrated mental health care system that enables clear shared planning for managing risk and relapse prevention. One example of this is the role of joint crisis plans (JCPs) in the United Kingdom. The CRIMSON study [24] was an individual-level RCT that compared the effectiveness of JCPs with treatment as usual (TAU) for people with a diagnosis of schizophrenia. There was no significant impact on the primary outcome (reduced coercion into hospital). It was noted that when faced with crisis, in spite of the considerable effort in developing the JCP with service users, the teams reverted to *custom and practice*. The staff did not consult JCPs in planning the team response to a crisis. Furthermore, people in receipt of services experienced an inability to influence clinicians’ behaviors, and this was interpreted as signaling a lack of respect for their views and opinions. Consequently, they described their interactions with clinicians as *playing the game*, that is, appearing to comply with treatment decisions. Clinicians themselves experienced their interactions with people using services as ritualized, especially in the context of responding to increased risk [25].

Gumley and Park [17] highlighted that relapse prevention based on EWS monitoring relies on the person receiving services initiating help seeking in the context of feeling vulnerable and threatened. Many individuals find help seeking a challenge and may have had difficult or traumatic experiences of psychosis. Delay in help seeking narrows the window of opportunity for successful relapse prevention, which in turn increases reliance on coercive measures confirming pre-existing negative expectations. It is therefore essential to develop and evaluate an intervention that can facilitate safe and honest disclosure of possible EWSs while also reorienting the response of mental health teams and the actions of their staff in a crisis toward a more collaborative approach.

**Figure 1:**
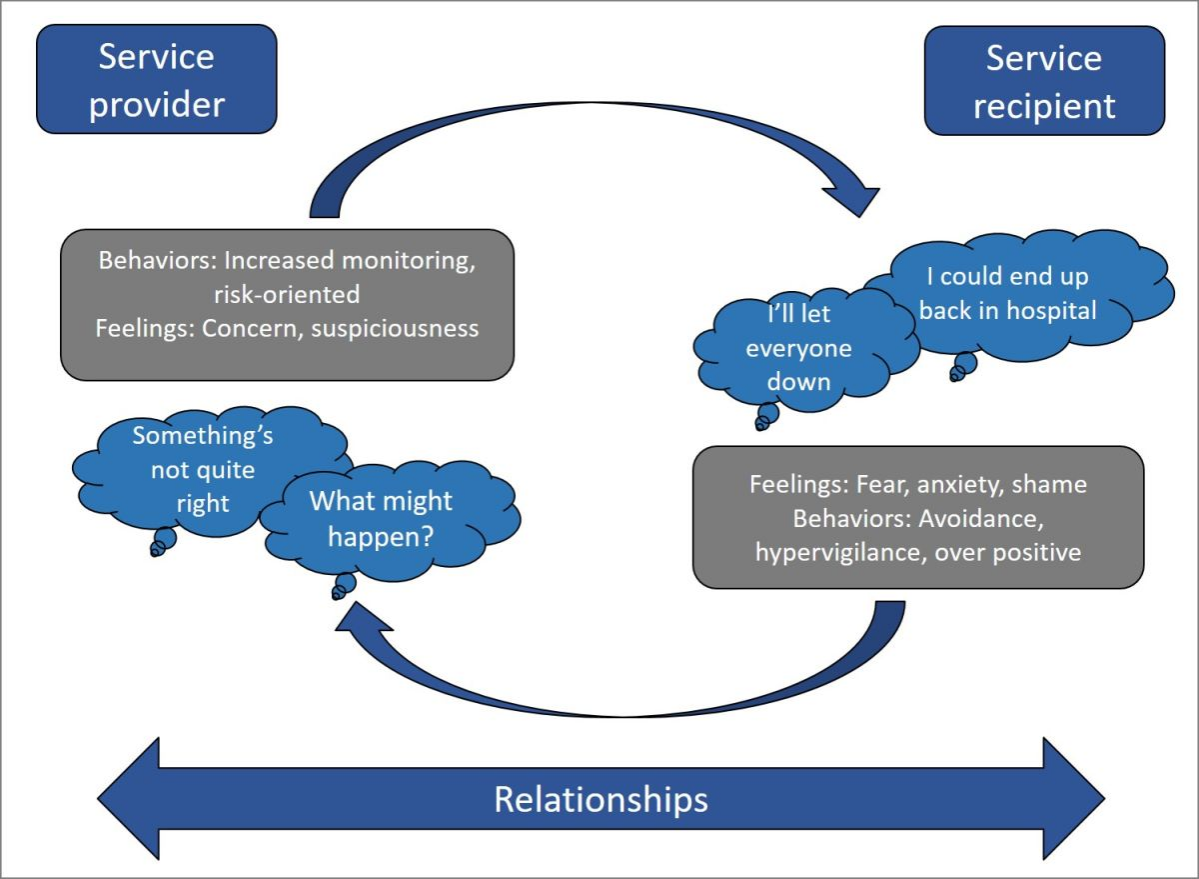
Cognitive interpersonal framework for early warning signs.

Our conceptual framework for improving relapse detection and prevention aims to understand how EWSs unfold in the context of important caring relationships. Figure 1 provides an illustration of our cognitive interpersonal model for EWSs. Fear of recurrence drives feelings of fear, anxiety, and shame. Coping strategies to regulate emotional distress (eg, increased hypervigilance, worrying, and avoidance) shape care providers’ own cognitive and emotional responses to perceived increased risk of relapse. For example, care providers may interpret increased emotional distress or avoidance (eg, cancelling appointments) as evidence of increased risk prompting changes in clinical care and risk management. These changes may further confirm individuals’ negative expectations of services and fear of recurrence. Therefore, interventions that can enhance positive emotional awareness (through self-monitoring), choice and autonomy (through self-management promotion), and improved communication (through increased understanding) could provide a means to disrupt and change negative interpersonal cycles.

Digital technology has the potential to offer a step change that can influence early signs monitoring both for people receiving and providing mental health services [26,27]. Mobile phones to support health care are promising for the delivery of interventions that are unconstrained by the limitations of existing treatment settings. Mobile phones are widely available and are continuously dropping in cost, and nearly 3 billion people are projected to own a mobile phone by 2020 [28]. Mobile phone ownership among people who experience psychosis is increasing with estimated rates of ownership from 66.4% (95% CI 54.1%-77.6%) rising to 81.4% (n=454) in more recent studies [29]. Furthermore, people with psychosis express an interest in the use of mobile phones to enhance contact with services and to support self-management [29,30]. Mobile phones, particularly *mobile phones*, offer opportunities for ecological momentary assessment (EMA) to collect data on repeated occasions, in real time, and in the context of daily life [31]. This enhances the ability to assess individuals in their usual context, temporally close to relevant events, and intensively and repeatedly as psychological processes unfold over time [32]. Bell et al [33] found that across 9 interventions (n=459), interventions for people with psychosis using EMA show satisfactory levels of feasibility and acceptability as well as preliminary evidence of improved clinical outcomes. Furthermore, studies that prospectively assess symptom course using mobile phone apps in people with psychosis show promise [34-45] in terms of feasibility of, acceptability of, and adherence to regular daily monitoring and also delivery of in-time interventions to support coping [36] or delivery of cognitive behavioral therapy [38].

However, studies employing mobile phones to promote relapse detection and intervention are not without their challenges. The Information Technology–Aided Relapse Prevention Program in Schizophrenia (ITAREPS) utilized a weekly text-based monitoring system to detect EWSs in people with psychosis. This system gathers data on early signs of psychosis using text messaging with clinicians notified by alerts where scores breach a specified threshold. Treating clinicians are expected to increase medication by 20% within 24 hours of an alert. In the initial studies [46,47], adherence by people using services and family members was problematic, in that only 47% were described as *high* users with 70% return rates. In the ITAREPS RCT [48], trial adherence improved; however, adherence to the treatment protocol was problematic at 39% largely owing to conflicts between protocol-indicated medication increases and psychiatrists’ clinical judgment. Komatsu et al [49] found that adherence to the ITAREPS protocol was improved when mental health nurses were involved in triaging EWS assessments. More recently, Eisner et al [50] demonstrated that over 6 months, adherence to mobile phone-based assessments of EWSs was 65% and successfully predicted increased psychotic symptoms 3 weeks later.

In sum, mobile phone technology offers a significant opportunity to deliver EMA-based monitoring of changes in well-being that are ecologically valid and contextually sensitive. Preliminary studies show encouraging levels of user acceptability and engagement, and thus, they offer the potential to recognize EWSs and activate timely assessment and intervention to promote self-management and relapse prevention. However, there are important implementation challenges to ensure that these technologies can enhance relationships and shared decision making, particularly during times of increased stress and crisis.

### Objectives

The overall aim of this study is to establish the feasibility of conducting a definitive cluster randomized controlled trial (cRCT) comparing Early signs Monitoring to Prevent relapse in psychosis and prOmote Well-being, Engagement, and Recovery (EMPOWER) against TAU. We will establish the parameters of the feasibility, acceptability, usability, safety, and outcome signals of an intervention as an adjunct to usual care that is easily deliverable in the National Health Service (NHS) and Australian community mental health service (CMHS) settings. The EMPOWER intervention aims (1) to enhance the recognition of EWSs by people using services and their carers and (2) to provide a stepped-care pathway that is either self-activated or in liaison with a carer and/or community health care professional, which then (3) triggers a relapse prevention strategy that can be stepped up to a whole team response to reduce the likelihood of a psychotic relapse.

Specifically, we aim to do the following:

1. Enhance and tailor our mobile phone software app to deliver EWS monitoring, self-management interventions, and access to a relapse prevention pathway that is firmly embedded in whole team protocols and action.
2. Determine rates of eligibility, consent, and recruitment of potentially eligible participants (people using services, carers, and care coordinators) to the study.
3. Assess the performance and safety of the EMPOWER Class 1 Medical Device (CI/2017/0039).
4. Assess the feasibility, acceptability, and usability of the intervention including feedback on suggested enhancements from people receiving the intervention, peer support workers, and clinicians.
5. Assess primary and secondary outcomes to determine preliminary signals of efficacy of the EMPOWER Relapse Prevention Intervention as a basis for the estimation of sample size requirements of a future definitive trial.
6. Undertake a qualitative analysis of relapses to refine the intervention in the main trial.
7. Establish the study parameters and data-gathering frameworks required for a coordinated health economic evaluation of a full trial across the United Kingdom and Australia.

## Methods

### Trial Design

**Figure 2:**
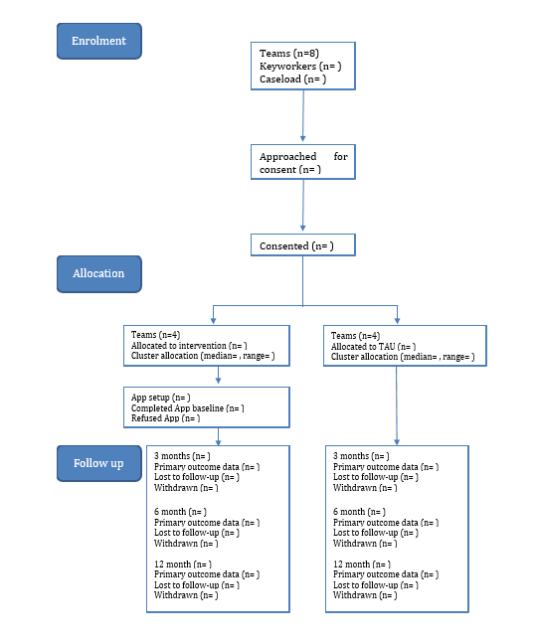
Consolidated Standards of Reporting Trials flow diagram. TAU: treatment as usual.

We will evaluate EMPOWER using a multicenter, 2-arm, parallel group cRCT involving 8 purposively selected CMHSs (2 in Melbourne, Australia, and 6 in Glasgow, Scotland) with 12-month follow-up. The CMHS will be the unit of randomization (the cluster), with the intervention delivered by the teams to people using services and with outcomes assessed within these clusters. The Standard Protocol Items: Recommendations for Interventional Trials checklist is provided as an additional file. The study is planned and implemented in concordance with the Consolidated Standards of Reporting Trials (CONSORT) cluster trial extension [51] and the extension to randomized pilot and feasibility trials [52]. We chose a cluster design as the EMPOWER intervention aims to enable a team-based response to people in receipt of services whose real-time EWS monitoring has activated a relapse prevention pathway. Our CONSORT diagram is detailed in Figure 2.

### Ethics and Governance

The West of Scotland Research Ethics Service (GN16MH271 Ref: 16/WS/0225) and Melbourne Health Human Research Ethics Committee (HREC/15/MH/344) approved the study. The study sponsors are NHS Greater Glasgow & Clyde in the United Kingdom and North Western Mental Health in Australia. Approvals from the NHS Health Research Authority and a notice of no objection for a trial of a medical device (CI/2017/0039) from the United Kingdom’s Medicines and Health care products Regulatory Agency (MHRA) was also received for the trial, and it has been prospectively registered (ISRCTN99559262). The basic trial methods of enrolment, interventions, and assessments are summarized in Table 1.

**Table 1:** Participant timeline.

Assessment timeline		Study period and time point				
		Enrolment (baseline)	Allocation (0 months)	Post allocation		Close-out (12 months)
				3 months	6 months	
**Enrolment**						
	Eligibility screen	X^a^	—^b^	—	—	—
	Informed consent	X	—	—	—	—
	Allocation	—	X	—	—	—
**Intervention**						
	EMPOWER^c^	—	—	X	X	X
**Service** **u** **ser assessments**						
	Feasibility	X	—	X	X	X
	Acceptability and usability		—	X	X	X
	Remission status	X	—	X	X	X
	Relapse		—	X	X	X
	Positive and Negative Syndrome Scale	X	—	X	X	X
	Personal and Social Performance Scale	X	—	X	X	X
	Calgary Depression Scale for Schizophrenia	X	—	X	X	X
	Time Line Follow Back	X	—	X	X	X
	Hospital Anxiety and Depression Scale	X	—	X	X	X
	Personal Beliefs about Illness Questionnaire	X	—	X	X	X
	Service Attachment Scale	X	—	X	X	X
	Medication Adherence Rating Scale	X	—	X	X	X
	EuroQol 5 Dimension	X	—	X	X	X
	Assessment of quality of life	X	—	X	X	X
	RUQ^d^	X	—	X	X	X
	Questionnaire for Personal Recovery	X	—	X	X	X
	Generalized Self Efficacy Scale	X	—	X	X	X
	Psychosis Attachment Measure	X	—	X	X	X
	Perceived Criticism and Warmth Measure	X	—	X	X	X
**Carer assessments**						
	Feasibility	X	—	X	X	X
	Carer Quality of Life 7 Dimension	X	—	X	X	X
	EuroQol 5 Domain 5 Level	X	—	X	X	X
	Resource Use Questionnaire	X	—	X	X	X
	Perceived Criticism and Warmth Measure	X	—	X	X	X
	Involvement Evaluation Questionnaire	X	—	X	X	X
**Care coordinator**						
	Feasibility	X	—	X	X	X
	Service Engagement Scale	X	—	X	X	X

^a^Item was applicable at the relevant study time point.

^b^Item was not applicable at the relevant study time point.

^c^EMPOWER: Early signs Monitoring to Prevent relapse in psychosis and prOmote Well-being, Engagement, and Recovery.

### Preliminary Work—Patient and Public Involvement

At the outset of the study, we conducted extensive consultation with key stakeholders including mental health staff (n=88), people with lived experience (n=21), and carers (n=40) in a series of 25-focus groups across Glasgow and Melbourne. The stakeholder consultation shaped the development of the EMPOWER intervention [53]. The Scottish Recovery Network also played a key role in shaping further consultation with people with lived experience in further refining the intervention development and planning.

### Eligibility Criteria

Participation will be sought from CMHS in NHS Greater Glasgow & Clyde in the United Kingdom and North Western Mental Health Services in Melbourne, Australia. All participants (mental health staff, service users, and carers) will be approached for their informed and written consent before assessment and randomization. Research assistants (RAs) will be responsible for recruitment and written informed consent. Recruitment took place between September 2018 and July 2019. Final follow-up assessments were completed end of June 2019.

### Community Mental Health Services

We will likely engage CMHSs to have 5 or more care coordinators willing to participate for a period of 12 months and where potential care coordinators having eligible service users on their caseload being likely to consider participation.

### Service Users

Service users from participating CMHSs are eligible for inclusion if they are adults (aged 16 years and above); are in contact with a local CMHS; have either been admitted to a psychiatric inpatient service at least once in the previous 2 years for a relapse of psychosis or received crisis intervention (eg, via a crisis intervention service, reengaged with a CMHS) in the previous 2 years for a relapse of psychosis; have received a diagnosis of schizophrenia-related disorder, specifically 295.40 schizophreniform disorder (International Classification of Diseases-10 [ICD10]=F20.81), 295.70 schizoaffective disorder (ICD10=F25), 295.90 schizophrenia (ICD10=F20.9), 297.10 delusional disorder (ICD10=F22); and are able to provide informed consent as adjudged by the care coordinator, if in doubt, the responsible consultant.

### Carers

Carers of people receiving support from participating CMHSs will be eligible for inclusion if they have been nominated by eligible participants; they are in regular contact with the person receiving services; and they provide informed consent to participate in the study.

### Exclusion Criteria

Individuals will be ineligible for participation if they do not meet the inclusion criteria outlined above. In addition, participants will be excluded if they have experienced a recent relapse operationally defined as having been discharged from the care of a crisis team or psychiatric inpatient service within the previous 4 weeks.

### Interventions

In describing the EMPOWER intervention in the following section***,*** we have utili***z***ed the Template for Intervention Description and Replication Checklist [54].

#### Early Signs Monitoring to Prevent Relapse in Psychosis and Promote Well-being, Engagement, and Recovery Intervention

##### Rationale

The rationale for the EMPOWER intervention was informed by the cognitive interpersonal model outlined in Figure 1 and has been designed to enable participants to daily monitor changes in their well-being. In EMPOWER, we refer to changes in well-being as *ebb and flow* as a means of moving away from risk-orientated monitoring that can sensitize individuals to increased fear of relapse. The terminology also conveys a normalizing framework for understanding changes in emotions and psychotic experiences in daily life. The EMPOWER intervention is blended with peer support. A peer support worker (PSW) is involved in setting up and personalizing the daily questionnaire, alongside regular fortnightly follow-up. Clinical triage of changes in well-being that are suggestive of EWSs is enabled through an EMPOWER algorithm that triggers a check in prompt (ChIP). This enables prompt identification of EWSs and triggers a relapse prevention pathway.

The EMPOWER app was developed through consultation with people using services, their carers, and mental health professionals. Service user participants have access to the EMPOWER app for up to 12 months of the intervention period. EMPOWER was developed as a flexible user-led tool to (1) daily monitor the *ebb and flow* of changes in their well-being which incorporates, (2) personalized EWS items, (3) enables the delivery of EMPOWER (self-management) messages directly to service users and, (4) provides a mobile phone user interface to enable service users to review their own data and keep a diary of their experiences.

##### Materials

Daily monitoring of well-being is initiated by pseudorandom mobile phone notifications to complete the EMPOWER questionnaire. The questionnaire contains 22 items reflecting 13 domains (eg, mood, anxiety, coping, psychotic experiences, self-esteem, connectedness to others, fear of relapse, and personalized EWSs). Items include both positive (eg, “I’ve been feeling close to others”) and negative (eg, “I’ve been worrying about relapse”) content. Each item is completed using a simple screen swipe that enables quick and efficient completion by users. Each item is automatically scored on a scale of 1 to 7. Where particular items score >3, users are invited to complete supplementary questions to enable a more fine-grained assessment of that domain. This provides up to a maximum of 56 questionnaire items. The questionnaire was piloted among 7 participants for an average of 36.7 days (range 32-49), and the app was completed on an average of 24.5 days (range 16-35), giving an overall rate of engagement of 68.9%.

##### Procedures

A PSW meets with participants on an individual basis to introduce the rationale for using the app, collaboratively sets up the app on their own or a study mobile phone, and supports the individual’s familiarization with the handset and app functions. Participants are invited to choose up to 3 personalized EWS items to be included in the EMPOWER questionnaire, and further personalization of delusion specific items can also be made. Where possible, an individual’s care coordinator and nominated carer are invited to contribute to this meeting. Participants are invited to undertake daily monitoring for an initial 4-week baseline period to help establish their personal baseline of the *ebb and flow* of their well-being. During this period, additional support is provided by PSWs through weekly telephone follow-up. This provides an opportunity to encourage usage of the app, solve any technical problems, and identify any adverse effects. At the end of the 4 weeks, a further meeting is arranged with a PSW or mental health nurse to review monitoring, encourage engagement with EMPOWER messages, agree participants’ preferences for actions in response to changes in well-being that are suggestive of EWSs and encourage continued utilization of local CMHSs for clinical care. All participants are offered ongoing fortnightly PSW support to encourage use of the app: to support their reflection on changes in well-being and their broader context including, for example, stressful life events, and to encourage use of self-management strategies prompted by EMPOWER messages.

##### Digital Procedures

**Figure 3:**
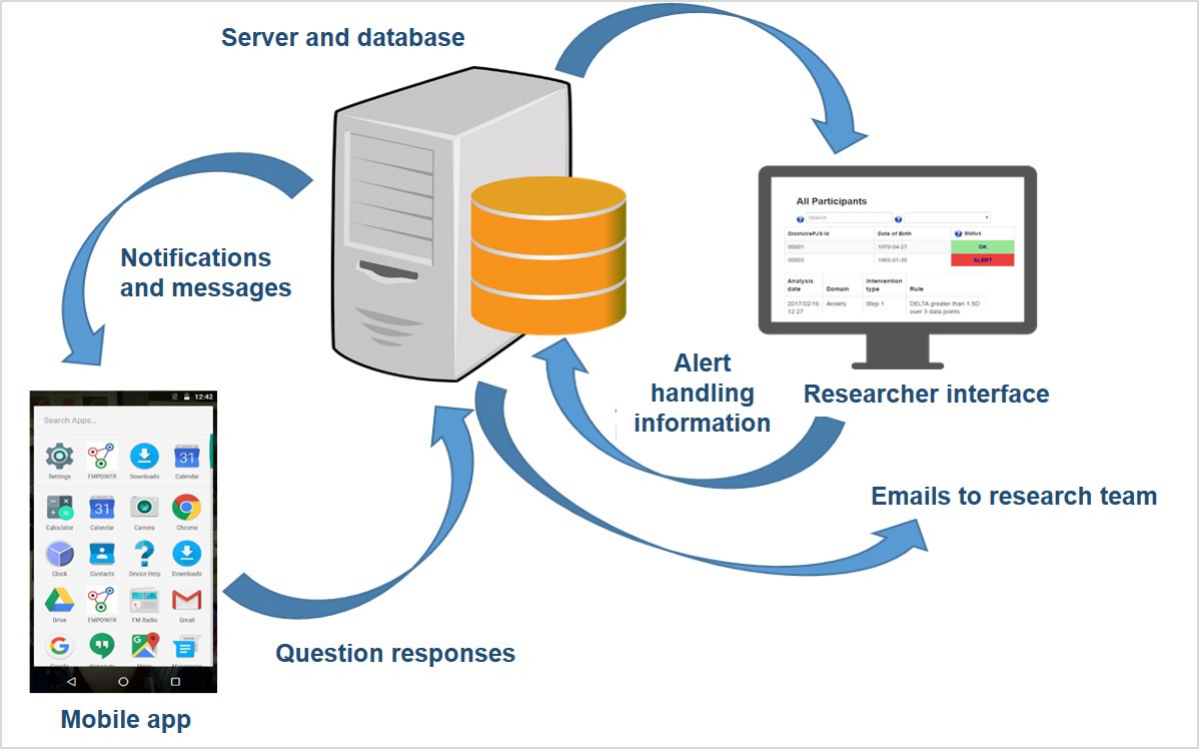
Early signs monitoring to prevent relapse in psychosis and promote well-being, engagement, and recovery system.

The analysis and handling of questionnaire data is governed by the EMPOWER algorithm. The EMPOWER algorithm is a Class 1 Medical Device (ISO 14155:2011[E]), which is an algorithm that forms one part of a broader system that is designed to identify and respond to changes in well-being that are suggestive of EWSs. Figure 3 provides a graphical representation of the system’s high-level components and data flow.

Participants use a mobile phone app that prompts them to answer a daily questionnaire about potential EWSs of psychosis. The data are then submitted to the EMPOWER server and analyzed by the EMPOWER algorithm. The algorithm compares a participant’s latest data entry against their personal baseline. If changes exceed predefined thresholds, a ChIP is generated for the participant. The consequences of the ChIP are that the research team, which includes a registered mental health nurse (in the United Kingdom), clinical psychologists (United Kingdom and Australia), and general psychologist (Australia only), is emailed about the participant and that the participant’s status is set to *alert* and is highly visible on a secure Web-based researcher interface. In addition to viewing and handling ChIPs, researchers can also view longitudinal graphs of their participants’ well-being and possible EWSs, filtered by question or by domain (group of questions). In response to ChIPs, a member of the research team checks in with the participant and based on the outcome of this triage assessment, they can share an update with the participants’ care coordinator who can, if indicated, escalate increased support to the participant from their local CMHS to reduce risk of relapse. These actions are agreed on a case-by-case basis, and we did not constrain the service user’s treatment provider to respond within a specific timeframe or indeed in a specific way. We have undertaken the responsibility to update the individual on any ongoing actions.

The EMPOWER algorithm also runs a separate process scan for EWS changes against the baseline. On the basis of these changes, the logic selects a message from the most appropriate of several content-based message pools (ie, one message pool contains helpful messages about *mood* and another about *anxiety and coping*). This message is delivered back to, and displayed on, the participant’s EMPOWER app. Messages are intended to help people have a greater sense of control over their mental health and well-being and to support self-management.

##### Training and Support to Community Mental Health Service Staff

Following randomization of CMHSs to EMPOWER, we will aim to provide training to mental health staff in those teams based on our model of relapse prevention, which emphasizes (1) therapeutic alliance, (2) barriers to help seeking, (3) familiarization with app, (4) developing an individualized formulation of risk of relapse, and (5) developing a collaborative relapse prevention plan. Following this, we aim to meet with care coordinators on a fortnightly basis to provide support in the implementation of EMPOWER. These meetings are aimed to clarify and encourage formulation of any changes and participants’ responses within the model and support clinicians to consider EMPOWER-consistent intervention options.

#### Treatment as Usual Control

TAU was chosen as a control condition in both the Glasgow and Melbourne centers as this provides a fair comparison with routine clinical practice. In Glasgow and Melbourne, secondary care is delivered by adult CMHSs, which largely involve regular, fortnightly, or monthly follow-up with a care coordinator and regular review by a psychiatrist. TAU is the modal comparison for digital interventions in schizophrenia. However, recent trials [38] have included active monitoring control groups. Our comparison with TAU will be reviewed before submitting the main trial application.

### Outcomes

All outcome measures will be administered at baseline and subsequently at 3, 6, and 12 months by RAs who will have been trained in the use of all the instruments and scales and who have achieved a satisfactory level of interrater reliability. RAs have as a minimum a strong honors degree in Psychology or a related discipline. Regular interrater reliability assessments will be conducted during the trial. RAs will enter anonymized participant data onto an electronic case record form hosted by the University of Aberdeen, with the exception of relapse data. Relapse data will be entered by the trial manager (SB). Data quality and error checking will be conducted at each time point including baseline, 3 months, 6 months, and 12 months post randomization.

#### Primary Outcomes

##### Feasibility

For all participants, outcome assessment will include the proportion of eligible and willing service users who then consent and the proportion continuing for 3, 6, and 12 months to the end of the study. We will report on the perspectives of service users, carers, and mental health staff in relation to the frequency of seeking help in relation to EWSs; the frequency in which a family member/carer has sought help in response to EWSs, and the frequency of clinical care that has changed in response to EWSs at 3, 6, and 12 months.

##### Acceptability and Usability

For those randomized to EMPOWER, we will report the length of time participants are willing to use the app and the number completing >33% of EWS datasets. We will also report the self-reported frequency of app use, frequency of sharing data with the keyworker, frequency of sharing data with the family member/carer, and frequency of accessing charts at 3, 6, and 12 months. We will also assess self-reported acceptability and usability using an adapted version of the Mobile App Rating Scale [55].

##### Safety—Adverse Events

Details of recording and reporting all adverse events is contained in our Standard Operating Procedure for Adverse Events that complies with Medical Devices Regulations 2002, ISO/FDIS 14155:2011 and Standards for Good Clinical Practice. An adverse event is defined as serious if it (1) results in death, (2) is a life-threatening illness or injury, (3) requires (voluntary or involuntary) hospitalization or prolongation of existing hospitalization, (4) results in persistent or significant disability or incapacity, or (5) is a medical or surgical intervention required to prevent any of the above, (6) leads to fetal distress, fetal death, or consists of a congenital anomaly or birth defect, or (7) is otherwise considered medically significant by the investigator. We will record any adverse device effects (serious or otherwise) arising from the EMPOWER algorithm. In addition, we will measure all adverse effects arising from study procedures including use of the EMPOWER app. We have prespecified (but not limited) these to (1) increased fear of relapse or paranoia associated with responding to questions in the EMPOWER app and (2) increased worries about surveillance by psychiatric services. We will also assess changes in fear of relapse using the Fear of Recurrence Scale [18].

The relationship between the investigational medical device and the occurrence of each adverse event will be assessed and categorized. In consultation with the RA and the trial manager, the chief investigator will use clinical judgment to determine the relationship. Alternative causes, such as natural history of the participant’s underlying condition, concomitant therapy, and other risk factors, will be considered.

We also record medical device deficiencies, which is any inadequacy of the EMPOWER medical device. These can include malfunctions, end user errors, and inadequate labeling.

##### Performance

The following performance endpoints have been identified:

1. Each participant has the app successfully uploaded on a mobile phone.
2. Each participant has personalized EWSs included in the EMPOWER questionnaire.
3. Each participant receives a daily prompt to complete their questionnaire.
4. Participants receive an EMPOWER message each time they complete the questionnaire.
5. Following 4 weeks of usage, the EMPOWER algorithm calculates each of the participant’s individualized baseline or variance of symptoms and experiences.
6. Participants can access charts of their symptoms and experiences covering 1-week and 1-month time intervals.
7. Following completion of the questionnaire, participants’ data are transferred to the secure server.
8. Researchers access participants’ questionnaire responses and generate charts to observe changes over time.
9. Researchers receive a record of alerts for each participant and can record actions in relation to these alerts.

#### Candidate Outcomes

##### Candidate Primary Outcome

Data related to symptom change and possible relapse will be extracted prospectively from electronic case notes by RAs. Relapse is defined as (1) a return or exacerbation in psychotic symptoms of at least moderate degree; (2) where symptoms have lasted at least one week in duration, and there is evidence of a decline in functioning or an increase in risk to self or others; and (3) there is evidence of a clinical response from services. Relapse criteria are summarized in Table 2.

**Table 2:** Early signs monitoring to prevent relapse in psychosis and promote well-being, engagement, and recovery relapse criteria.

Criteria	Notes and definitions
A return or exacerbation in psychotic symptoms of at least moderate degree; If present score=1	These are defined as first rank psychotic symptoms including hallucinations, delusions, thought disorder and persecutory paranoia In line with Positive and Negative Syndrome Scale assessments, moderate severity means that these occur at least occasionally or intrude on daily life to a moderate extent
*AND* Where symptoms have lasted *at least* 1 week in duration; If present score=1	There was clear evidence that duration of psychotic symptoms occurred over at least 1 week
*AND* Where there is evidence of a decline in functioning; If present score=1	Includes a decline in one or more of the role performance areas identified from the Personal and Social Performance Scale: Socially useful activities, including work and study (this should include cooperation with household tasks, voluntary work, and *useful* hobbies, such as gardening) Personal and social relationships (this includes relationships with a partner or relatives and broader social relationships) Self-care (personal hygiene, personal appearance, and dressing) General domains to consider are physical and psychological health care; lodging (area of residence and living space care); contribution to household activities; participation in family life or residential/day-center life; intimate and sexual relationships; childcare; social network, friends, and helpers; general interests; financial management; use of transport; coping skills in crisis; keeping social rules
*OR* An increase in risk to self or others; If present score=1	Increase in risk to self includes deliberate self-injury and/or suicidal ideation that was clinically significant in the investigator’s judgment. Evidence is required of either an increase in thoughts or an intent to act upon such thoughts. These must occur within the context of the episode and be accompanied by a service response. The service response can be reflected in that there is a statement of increased risk, there is a note of discussing safety plans, or staff have ensured that the participant has access to crisis contacts Increase in risk to others includes significant violent and aggressive behavior. This also includes homicidal ideation, with evidence of intent to act upon this. Violent and aggressive behavior should only be recorded as an increase in risk where there is evidence of a service response to manage this behavior
*AND* There is evidence of a clinical response from services; If present score for each of these criteria=1 (Maximum=3)	An increase or change in medication, increased home visits, or referral to crisis services Any hospital admission or imposition of a Community Treatment Order in response to psychosis Use of the mental health act to enforce an involuntary hospital admission

RAs who are not blinded to the treatment condition extracted data from electronic case records to document all recorded episodes of changes in psychotic symptoms, functioning, risk, and clinical management. These episodes will provide the basis for individual anonymized case vignettes that are submitted to our independent and blinded adjudication panel. All vignettes will be fully anonymized and any information relating to the EMPOWER intervention will be concealed. This panel will contain expert clinicians/researchers who will have the necessary knowledge, experience, and skills to make independent blinded judgments regarding relapse/exacerbation. The panel will determine that a relapse event has occurred and will also rate the severity of relapse on a 7-point scale based on the criteria included in Table 2. We will report time to first relapse, type of relapse (Relapse, Exacerbation, and Unspecified). We will also report number (%) with (1) return or exacerbation in psychotic symptoms, (2) duration of at least one week, (3) reduction in functioning, (4) increase in risk, (5) change in clinical management, (6) admission to hospital, and (7) use of mental health act. We will report details of all relapse outcomes across both groups. All participants will be assessed at each follow-up point for the presence of any of these criteria described in Table 2, enabling us to calculate a mean severity score across participants and allocated groups at each follow-up point.

##### Candidate Secondary Outcomes

We will also assess changes in symptoms, substance use, emotional distress, carer burden, service engagement, and adherence and health-related quality of life.

1. Mental health status: The Positive and Negative Syndrome Scale [56], Personal and Social Performance Scale [57], and the Calgary Depression Scale for Schizophrenia [58] will be completed with service user participants.
2. Substance use measures: Time line follow back for drugs and alcohol [59]
3. Emotional distress: Hospital Anxiety and Depression Scale [60] and the Personal Beliefs about Illness Questionnaire-Revised [61].
4. Service engagement: The Service Attachment Scale [62] and the Medication Adherence Rating Scale [63] will be completed by service user participants.
5. Health Economics: EuroQol 5 Dimension 5 Level (EQ-5D-5L) [64] and the Assessment of Quality of Life—8 Dimension (AQoL-8D) [65], the CarerQoL [66], and a Resource Use Questionnaire (RUQ).

##### Candidate Mechanisms

Measures have been selected that map directly onto hypothesized mechanisms of change and known predictors of relapse. Mechanisms of service user benefit are operationalized as improvements in personal recovery, empowerment, and utilization of social support.

1. Recovery and self-efficacy: Questionnaire for Personal Recovery [67] and the General Self-Efficacy Scale [68] will be completed by service user participants.
2. Social and interpersonal context: Psychosis Attachment Measure [69] and Perceived Criticism and Warmth Measure adapted from the Perceived Criticism Measure (PCM) [70] will be completed by service user participants.

#### Carer Outcomes

The Involvement Evaluation Questionnaire [71] will be completed as a measure of carers worrying, tension, urging, and supervision. A carer PCM adapted from the PCM described above will be used as a measure of carers’ perspectives on relationship quality.

We will also assess Carer Health Economic Outcomes using a purposively designed RUQ, time cost questionnaire, the EQ-5D-5L, and the CarerQol-7D.

#### Care Coordinator Outcomes

Participants care coordinators will complete the Service Engagement Scale [72].

### Process Evaluation

In line with recent MRC Guidance on process evaluation of complex interventions [73], we will produce a logic model for the EMPOWER intervention and conduct a process evaluation. The process evaluation will be used to explore the ways in which EMPOWER may operate to produce outcomes. Specifically, it will focus on intervention fidelity, exposure, reach, context, recruitment, retention, and contamination, as well as the acceptability of study procedures. We will interview service users, carers, mental health staff, and research staff to ensure a multiperspective understanding of the intervention. We will publish the protocol for the process evaluation elsewhere.

### Sample Size

No formal sample size calculation is appropriate for this pilot phase. The proposed sample size of up to 120 service users across 40 care coordinators in 8 CMHSs is deemed to be sufficient to establish feasibility and obtain parameters (including the relevant ICCs for the cluster design) to inform the design and size of a future definitive, pragmatic, multicenter, and multinational cRCT.

### Recruitment and Randomization

The unit of randomization is the CMHS (the cluster). Participating CMHSs will be randomized within stratified pairs to the EMPOWER Relapse Prevention Intervention or to continue their usual approach to care. A statistician at the Centre for Healthcare Randomised Trials (CHaRT) at the University of Aberdeen will provide the allocation codes. The 2 clusters in Australia form a single stratum. The 6 clusters in Glasgow will be paired based on similarity of catchment area in terms of social deprivation or CMHS type (eg, early intervention service).

Researchers will approach each eligible care coordinator and seek their consent to participate in the trial. Before randomization, consenting care coordinators will provide an anonymized list of their current potentially eligible caseload of people using services. This list will then be randomly ordered by CHaRT. Researchers will then approach identified people sequentially in blocks of up to 5 potentially eligible participants and seek informed consent to participate in the study. If there are further participants eligible for inclusion at the end of this block, the researcher will move onto the next block of 5 (if applicable). Care coordinators will provide participants with an easy-to-read information leaflet about the study to enable potential participants to express interest in finding out more information. In Australia, information posters will be displayed within staff areas of participating sites to inform care coordinators of the study and provide contact details of RAs, should they wish to participate.

We aim to approach and consent on an average of 3 participants per care coordinator (giving a total of up to 120 potential participants). Following consultation with the independent Data Management and Ethics Committee (DMEC) and the Study Steering Committee (SSC) in May 2018, these recruitment objectives were amended to 86 participants. This was because of a number of challenges to recruitment including (1) temporary suspension of the study to apply for registration with the MHRA, which delayed start up to recruitment, (2) comprehensive and detailed screening of all potential trial participants, (3) delays arising from care coordinators approaching potential participants, and (4) high rates of turnover among care coordinators. Following independent methodological and statistical advice, there was clear guidance that the original feasibility aims will be met by the trial, and this change to recruitment targets was approved by the funders. After completing baseline assessments on all consenting service users in care coordinators’ and CMHSs’ caseload, the Clinical Trials Unit (CTU) at CHaRT will conduct randomization of the CMHSs. For Australia, with just 2 clusters, this will be by simple randomization by the CTU. For Glasgow, with 6 clusters, the CTU will create 3 pairs of teams based on similarity of the catchment area in terms of social deprivation (Carstairs) score or CMHS type (eg, early intervention service). The CTU will randomly allocate one member of the pair to the intervention, and the remaining member will be allocated to control.

We will explore in this pilot phase the best method of randomly allocating the clusters in the full trial, specifically to establish what matching factors (if any, and/or if matching at all is appropriate, methodologically) are suitable. Any violations of the study protocol will be recorded and reported to the Research Ethics Committee, SSC, and the independent DMEC.

### Statistical Analysis

A full statistical analysis plan will be written before (and published on the CHaRT website) any analysis is being undertaken. All analyses will be carried out using the intention-to-treat principle with data from all participants included in the analysis including those who do not complete the intervention. Every effort will be made to follow up all participants in both arms for research assessments. The analysis will follow the guidelines of the CONSORT statement for clustered randomized trials and recommendations for the analysis of clustered randomized trials when presenting and analyzing the data. Here, we have potentially repeated measures on individual patients nested within care coordinators who are nested within teams (the unit of randomization) who are in turn nested within region (Australia and the United Kingdom or possibly to be known as Scotland). The analysis will adjust for these factors using appropriate random (service user, if relevant; care coordinator; and team) and fixed (region) effects. The trial statistician will remain blind until the main analyses are complete. Baseline characteristics of the study population will be summarized separately within each randomized group. Baseline characteristics will also be presented for drop-outs and completers within each treatment group.

A full Health Economic Statistical Analysis Plan will be written before any analysis is being undertaken. As part of the within trial economic evaluation, we propose to test 2 health-related quality of life measures (which can be used to assess Quality-Adjusted Life Years), the EQ-5D-5L, and the AQoL-8D in the feasibility trial. Although the EQ-5D-5L is very commonly used in the United Kingdom and Australian context, its sensitivity and appropriateness in people with schizophrenia has been seriously questioned [74]. The AQoL-8D is a newer HRQoL measure and was developed to be sensitive to the domains of quality of life that are important to people with mental health problems. A RUQ to capture costs incurred will also be tested. This questionnaire will need to be appropriate to both the United Kingdom and Australian context but may require some system-specific modules for services, which differ between the 2 settings.

### Data Sharing

Data generated by this research will be made available as soon as possible based upon reasonable request to the study chief investigator.

### Ethics Approval

West of Scotland Research Ethics Service (GN16MH271 Ref: REC/16/WS/0042) and Melbourne Health (HREC/15/MH/344).

## Results

Recruitment to the trial began in September 2017, and follow-up of participants was completed in July 2019. The database lock was in July 2019, followed by analysis and disclosing of group allocation.

## Discussion

Relapse among people with a diagnosis of schizophrenia is a major contributor to poorer outcomes in terms of greater symptom severity, distress, and risk of a range of adverse outcomes including coercion into care, self-harm, and suicide [8-12]. People diagnosed with schizophrenia worry about relapse, and this is linked to feelings of anxiety, shame, depression, and shorter time to relapse [18]. Relapse contributes to carers’ experiences of distress and worry and has been linked to greater needs for continuity of communication with mental health care professionals [75]. Mental health staff are concerned about the impact of relapse on service users and carers, but the impact on their roles are also considerable in terms of responding to crises and increased risk [24]. These sources of strain place relationships under significant pressure, which can break down communication, anticipatory care, and shared decision making especially in the context of EWSs of relapse. This can potentially result in loss of collaboration, increased risk of disengagement, and greater use of coercion, thus confirming many of the fearful and threat-based expectations held by service users, carers, and, arguably, the staff [25].

On the basis of our theoretical model outlined earlier, we have developed an intervention that blends a digital intervention with peer support, with triage of increased risk of relapse to prompt early interventions that are attuned to needs of service users. Our approach uses digital technology to enable service users to monitor the *ebb and flow* of emotional well-being in daily life and utilizes an algorithm to deliver messages to enhance attunement, awareness, curiosity, and self-management in response to changes in well-being. In our theory, the blending of digital with peer support is important to cultivate helpful conversations to promote greater autonomy, empowerment, and recovery. We anticipate that conversations focused around the *ebb and flow* of changes in well-being in daily life can also increase opportunities for earlier help seeking, collaboration, and shared decision making in the event of possible EWSs facilitated by clinician triage into a relapse prevention pathway embedded in mental health services.

The EMPOWER trial will deliver the protocol for a blended digital intervention and will establish the feasibility of running a definitive cRCT. We will establish the safety, acceptability, usability, and performance parameters of the EMPOWER intervention. We will learn how to optimize the identification, consent, and follow-up of potentially eligible participants. We will establish the feasibility of measuring and collecting our candidate primary, secondary, and mechanistic outcomes. However, in its own right, our study has a number of important broader implications for digital health interventions for people diagnosed with schizophrenia and psychosis. Our mobile phone app will be available for people to use for up to 1 year on a daily basis. To our knowledge, this will be the longest RCT of a mobile phone app in psychosis to date. In addition, the algorithm that underpins the assessment of *ebb and flow* is, to our knowledge, the first mental health app to be registered as a medical device trial (CI/2017/0039) by the United Kingdom’s Medicines and Healthcare products Regulatory Agency.

We will also establish and report the rater reliability of our a priori definition of relapse that has been informed by our theoretical model [18] that underpins the intervention. In studies of relapse in schizophrenia, there is a lack of consensus as to the definitions of relapse measurement [76], very often studies actually fail to report their definitions of relapse [77], and, to our knowledge, none have ever consulted people with lived experience of psychosis on how relapse is measured and defined. Our approach to relapse assessment aims to address these major shortcomings in the literature making the theory, rationale, measurement, and interrater reliability of our definition explicit. In addition, our approach will enable us to report time to relapse, severity of relapse, and mean severity scores across groups at each follow-up timepoint.

## Appendix

Multimedia Appendix 1 Peer-reviewer report from the National Institute for Health Research - Health Technology Assessment Programme (NIHR-HTA).

## Abbreviations

AQoL-8D: Assessment of Quality of Life—8 Dimension
CHaRT: Centre for Healthcare Randomised Trials
ChIP: check in prompt
CMHS: community mental health service
CONSORT: Consolidated Standards of Reporting Trials
cRCT: cluster randomized controlled trial
CTU: Clinical Trials Unit
DMEC: Data Management and Ethics Committee
EMA: ecological momentary assessment
EMPOWER: Early signs Monitoring to Prevent relapse in psychosis and prOmote Well-being, Engagement, and Recovery
EQ-5D-5L: EuroQol 5 Dimension 5 Level
EWS: early warning sign
ICD10: International Classification of Diseases-10
ITAREPS: Information Technology–Aided Relapse Prevention Program in Schizophrenia
JCP: joint crisis plan
MHRA: Medicines and Healthcare products Regulatory Agency
NHS: National Health Service
PCM: perceived criticism measure
PSW: peer support worker
RA: research assistant
RCT: randomized controlled trial
RR: risk ratio
RUQ: Resource Use Questionnaire
SSC: Study Steering Committee
TAU: treatment as usual
