# Supplementary material for: Early Signs Monitoring to Prevent Relapse in Psychosis and Promote Well-Being, Engagement, and Recovery: Protocol for a Feasibility Cluster Randomized Controlled Trial Harnessing Mobile Phone Technology Blended With Peer Support
Source: JMIR Res Protoc. 2020 Jan 9;9(1):e15058. doi: 10.2196/15058 (PMC6996736; doi:10.2196/15058)
Supplement: Multimedia Appendix 1 [file resprot_v9i1e15058_app1.pdf]

RESEARCH PROPOSAL EXTERNAL REVIEW OPEN COMMENTS

Review Questions

Question	Response
**Importance, acceptability and potential impact of the proposed research?**<br>Importance, acceptability and potential impact of the proposed research?	The grant application clearly states how important this research would be, especially if the aims of using Early Warning Signs (EWS) as a predictor of psychosis relapse could be linked to meaningful responses from healthcare teams which were valued by service users.
**Research methods, recruitment, and scientific quality?**<br>Proposed research methods, recruitment and scientific quality?	This grant application covers 6 separate work packages. The comments made in answer to this question relate principally to WP1, WP2 and WP4.<br><br>In WP1 and WP2 the research team plan to utilise focus groups as the main method of investigation.This would appear to be an appropriate method to meet the intended objectives. However, there remain some concerns. No details were provided regarding the likely composition of focus groups and this could have a baring upon the outcome. For example in WP1 there is an intention to recruit both service users and carers, would there be separate focus groups for each participant category? Having service users and their respective carers attending the same group might result in either participant becoming inhibited regarding the contributions they make. A similar issue could be present in WP2 with staff of differing grades being present at the same focus group, leading to junior staff feeling inhibited by the presence of senior staff.<br><br>A further concern regarding WP1 was the intended method of recruitment. Principally key workers would initially identify and approach service users to participate in the study. This could lead to biased recruitment in that only service users deemed to be 'suitable' would be invited. Other, more open, methods of recruitment, should be considered, e.g. direct advertising of the study at patient centres.

Question	Response
	The framework analysis described in both WP1 and WP2 is a further source of concern. Whilst recognising framework analysis as a valid technique in qualitative research the reliance placed by the research team on expedience and use of an apriori informed analysis jeopardises the intrinsic benefits of a qualitative approach to research.<br><br>WP4 is a pilot cluster controlled study. The methodology suggested for this work package appeared to be appropriate for the stated aims.
**Research team, resources and research management?**<br>Research team, resources and research management?	This is a complex, multi-centred, multi intervention study. An extensive research team is set out, with indication as to how members contribute to the necessary overall skill mix.
**Ethics and governance?**<br>Ethics and governance?	There were no undue ethical or governance concerns raised by the grant application
**Patient and public involvement?**<br>Patient and public involvement?	There would appear to be strong involvement of patient and public groups in this research proposal. Specific mention is made of Scottish Recovery Network (SRN) as active collaborators in both the research proposal and the design of the EMPOWER relapse prevention intervention.
**Research outputs?**<br>Research outputs?	A clear strategy is in place detailing how the research finding will be disseminated to service users, enabling them to gain from the study. As this is an initial, pilot study, then a further key dissemination route is to mental health teams, with an aim to ensure further recruitment and participation for the intended full study.
**What are the key strengths of this proposal?**<br>What are the key strengths of this proposal?	The range of planned work packages to address the research question from a range of perspectives. Furthermore the desire of the research team to link early detection of relapse to meaningful, non-threatening and empowering interventions which aim to prevent a full relapse from occurring.
**What are the key weaknesses of this proposal?**<br>What are the key weaknesses of this	Although not perhaps a weakness, rather a risk.<br>Several of the planned work packages (WP1, WP3 WP4) have a focus upon evaluating or assessing the acceptability and feasibility of a planned intervention. Although it is not clear to what extent one work package

Question	Response
proposal?	is reliant upon the successful completion of a preceding package, there still remains a risk that later work packages may be affected by the findings of an earlier one.<br><br>A further concern regards the proposed smart phone application. It is not clear how much development work is still needed on this app. In parts of the grant application it seems as if this app is already functioning and hence the objective is to evaluate it. In other parts of the grant application there is an emphasis on the need to enhance and modify the app. There is a concern therefore that this study could be delayed by the need to make and re-make modifications to the proposed app.
**Questions for the applicants**<br>Do you have any questions for the applicants that you would like the opportunity for the applicants to respond to prior to the proposal being considered by the funding board?	None

National Institute for Health Research

RESEARCH PROPOSAL EXTERNAL REVIEW OPEN COMMENTS

Review Questions

Question	Response
**Importance, acceptability and potential impact of the proposed research?**<br>Importance, acceptability and potential impact of the proposed research?	This piece of research is very timely and relevant to the call. The use of smartphone apps is becoming more and more popular and a potentially cost efficient way of handling this type of monitoring for EWS.
**Research methods, recruitment, and scientific quality?**<br>Proposed research methods, recruitment and scientific quality?	The research methods are relevant to the study. The approach to recruitment for the pilot trial is sensible and the applicants have considered the potential issues that could arise.<br><br>While a comparison to TAU is normal for this type of study there was little information in the application about how the variability of the control treatment would be recorded and handled.<br><br>One section of the analysis appeared to be very through for a pilot study sample, I'm sure with such an experienced team though results from this type of analysis would not be over reported
**Research team, resources and research management?**<br>Research team, resources and research management?	The research team are very experienced and appear well positioned to carry out this piece of research.<br><br>I'm not convinced of the benefit of having centres in Australia, given that potentially TAU could look very different there and this may influence the apps development. Research management is also further complicated by being across two nations.<br><br>In terms of development of the app, the potential for issues to arise during the development of such smart apps and I wonder if the team has enough leeway within the timelines to accommodate any delays.
**Ethics and governance?**<br>Ethics and governance?	Ethics and governance have been covered adequately within the application

Question	Response
**Patient and public involvement?**<br>Patient and public involvement?	PPI has been appropriately considered as without this input the app developed will not be feasible or of use to the demographic.<br><br>Careful consideration of the demographic needs to be considered and sensible PPI will include this as the older generation may be less open to using such an intervention. Therefore age may be very important variable to consider in any analysis.
**Research outputs?**<br>Research outputs?	Research outputs have been considered and are appropriate for this study
**What are the key strengths of this proposal?**<br>What are the key strengths of this proposal?	Development of smart apps are very timely and potentially a great source of intervention for use in many disease areas. This study is aimed at developing and starting the testing of such an app. Plenty could be learned about development of these apps from such a study.
**What are the key weaknesses of this proposal?**<br>What are the key weaknesses of this proposal?	Description of the data recording for the TAU arm of the trial. Despite this I feel that this application is very strong.
**Questions for the applicants**<br>Do you have any questions for the applicants that you would like the opportunity for the applicants to respond to prior to the proposal being considered by the funding board?	No further questions to ask

RESEARCH PROPOSAL EXTERNAL REVIEW OPEN COMMENTS

Review Questions

Question	Response
**Importance, acceptability and potential impact of the proposed research?** Importance, acceptability and potential impact of the proposed research?	The topic is of central importance to psychiatric services, in which much of the resource (and hence the cost) is dedicated to the management of psychosis. The episodic nature of psychosis is responsible for much of the burden to those who suffer from it, their carers, and the community at large, which is responsible for providing the necessary services. The symptoms of psychosis are extremely heterogeneous, and include many symptoms that lie outside the central syndrome. Many of these ancillary symptoms become more prominent very early in the process of relapse, hence early warning symptoms. For this reason, the best hope for improving the management of psychosis lies in a close evaluation that leads to a personalised treatment strategy. In particular, the identification of affective mechanisms underlying exacerbation and relapse is likely to point to personalisation. The proposal by Gumley and colleagues offers the prospect of developing just such a strategy. I like the relatively novel approach of using smartphones to assist in the personalisation of treatment. In general people with psychosis welcome the respectfulness of treatment plans which address their specific concerns, and the effectiveness of such treatment is likely to be enhanced by its acceptability. I therefore think that the potential impact of this proposal is considerable, in terms of both effectiveness and cost effectiveness.
**Research methods, recruitment, and scientific quality?** Proposed research methods, recruitment and scientific quality?	I had no particular criticisms of the methodology, which is of a high standard, and will answer the important question addressed by the research. The use of work packages means that the programme of research is well planned, and this increases the likelihood of effective delivery. I wondered about the allocation of ten months to WP2, which seems a bit long, but it is good to have some potential leeway in the system. Cluster randomisation requires diligent management on the part of the research team, in particular in keeping recruitment levels on track, but the research team

Question	Response
	seems to have the capacity to be able to do this. Cluster randomisation is clearly the only approach that effectively captures the delivery of a treatment at the level of clinical teams.
**Research team, resources and research management?** Research team, resources and research management?	I thought this was impressive, and should be able to deliver on the project.
**Ethics and governance?** Ethics and governance?	I did not think the project raises serious ethical issues. The applicants are well aware of the issues and specifically mention the potential negative consequences of early signs monitoring and the difficulties that may emerge in relapse prevention strategies. This should enable them to minimise problems
**Patient and public involvement?** Patient and public involvement?	This was extensive and central to the development of the project, given its specific joint planning of therapy with clients.
**Research outputs?** Research outputs?	In addition to the scientific outputs in terms of research papers, the applicants have a sizeable programme of public dissemination.
**What are the key strengths of this proposal?** What are the key strengths of this proposal?	The key strength is that it involves therapists and clients in a joint project of identifying problems, and working out ways of dealing with them. This way of involving case managers in personalising treatment carries the possibility of circumventing the almost universal failure of implementation programmes based on training staff in new techniques of management and treatment. The failure arises because case managers are trained, and then fail to use the training to deliver the treatments (because they are difficult, because they have other priorities that they may thus favour). The use of smartphones is an elegant way of increasing acceptability and delivery.
**What are the key weaknesses of this proposal?** What are the key weaknesses of this	I did not identify any key weaknesses.

Question	Response
proposal?	
**Questions for the applicants**<br>Do you have any questions for the applicants that you would like the opportunity for the applicants to respond to prior to the proposal being considered by the funding board?	They might say why they allocated 8 months to stage WP2

National Institute for Health Research

RESEARCH PROPOSAL EXTERNAL REVIEW OPEN COMMENTS

Review Questions

Question	Response
**Importance, acceptability and potential impact of the proposed research?**<br>Importance, acceptability and potential impact of the proposed research?	There is a clear reason for doing this research - its proposed use of a mobile phone app is an innovative one that could benefit many greatly, providing them with a new means of managing their illness.<br><br>I have not heard of any similar kind of research.
**Research methods, recruitment, and scientific quality?**<br>Proposed research methods, recruitment and scientific quality?	In general I would be happy for myself or a loved one to take part in this research, albeit with some provisos. I feel that some acknowledgement needs to be made of the sensitivity with which service users need to be communicated with in introducing such a project to them. Perhaps some further training of, e.g. the peer support workers involved, is necessary to deal with potential difficulties, such as:<br><br>- the problems randomisation can cause, with some unhappy at being in the TAU group.<br>- the paranoia that being a part of a study can cause in some service users.<br>- the paranoid fear of technology that is common.<br>- the complexity of using an app that may be too testing for some.<br>- exaggerated hopes of recovery.<br><br>I feel the research is probably likely to be deliverable, though there could be some difficulties in motivated time-constrained staff.<br><br>I feel the research methods are generally appropriate, and any difficulties arising could be managed.
**Research team, resources and research management?**<br>Research team, resources and research	N/A

Question	Response
management?	
**Ethics and governance?**<br>Ethics and governance?	There is some potential for harm to participants surrounding the issue of whether it is more efficacious for a service user to be focusing on the way he/she is feeling, or to spend more time taking their mind off themselves.<br><br>But we don't have a conclusive answer to this yet, so think any risk included here is necessary, and probably a mild one, providing those involved are qualified in judging whether consent is informed or not.
**Patient and public involvement?**<br>Patient and public involvement?	I welcome the use of service users such as the peer support workers involved, and those in the group activities. I would have liked to have seen further service user involvement in the more senior positions.<br><br>The costs seem to have been considered.
**Research outputs?**<br>Research outputs?	I feel dissemination could be broader. There will be a lot of interest in the outputs of this project, and therefore why not utilise PDFs, a further app, or perhaps even the likes of Mind or Rethink websites to promote a link to the output information. Indeed, for those who struggle to read at length, a short video on Youtube could be employed.
**What are the key strengths of this proposal?**<br>What are the key strengths of this proposal?	- Innovative use of technology.<br>- Provides an instantaneous means of management of early warning signs.<br>- Could potentially be utilised by wide spectrum of service users.<br>- Service user involvement present.<br>- Carers allowed to participate, and their wellbeing assessed as well as service users'.
**What are the key weaknesses of this proposal?**<br>What are the key weaknesses of this proposal?	- The care pathway at present seems a bit vague.<br>- Outputs could be more widely disseminated.<br>- Much is dependent on staff motivation - this is not a given.<br>- Communication skills over sensitive areas regarding participation will have to be attended to in more depth.<br>- Over-dependency on the app doesn't seem to have been considered, along with effects of withdrawal.
**Questions for the**	- Do you not think £20 for participants' time and in put

Question	Response
**applicants**<br>Do you have any questions for the applicants that you would like the opportunity for the applicants to respond to prior to the proposal being considered by the funding board?	during WP1 is a rather cursory sum?<br><br>- Will signposting be included in service users' care pathways - e.g. reminders of the Samaritans',or Sanelines' phone numbers?<br><br>- Do you think the elderly may be under represented in this project?

National Institute for Health Research

RESEARCH PROPOSAL EXTERNAL REVIEW OPEN COMMENTS

Review Questions

Question	Response
**Importance, acceptability and potential impact of the proposed research?** Importance, acceptability and potential impact of the proposed research?	The importance is clearly stated. The study has plans to address acceptability in the development of the app for EWS but I didnt see any qualitative work being included in the actual feasibility trial itself. I suggest that given this is a feasibility study that the process data should be collected from the participants (and i assume that there is a two level of participants- staff who are in the CMHT and the service users recruited from within this). If the app is shown to be acceptable and useful, then the potential impact could be great for EWS and prevention of relapse in schizophrenia.
**Research methods, recruitment, and scientific quality?** Proposed research methods, recruitment and scientific quality?	I think it is useful to have an international collaboration with Australia. however, I question whether it is useful to demonstrate feasibility in only one service within each country. I would suggest that it is useful to involve more than one organisation within each country. Often it is easier to demonstrate feasibility in a service that may be well set up to undertake the research. I would like to see a further justification of only using one service within each of the countries from the applicants.<br><br>The study seems to contain 2 levels of intervention - the app and the training for CMHT staff. There is no mention of how this training will be evaluated. ie how will you measure that the workers now have the required capabilities to work with the EWS APP intervention? In addition, it is not clear if fidelity measures for the CMHT staff will be collected.<br><br>How will the analysis account for the cluster effect of team but also cluster effect of clinician? The issue may be that some CMHT staff may be better at engaging and implementing an alliance this enhancing the effect of the app. This is an issue that should be investigated within the feasibility study and accounted for in the analysis. There is also some important feasibility issues of "whole team training" and how many of the team would be a critical mass? It is not clear how they

Question	Response
	would ensure that the case manager of participants would definitely have the training (they may have been absent during the training for example). there is a lack of clarity regarding the recruitment of the case managers, and how to ensure they participate in the training. In addition, what is the justification for whole team training when you only intend to recruit 3-5 staff per team?
**Research team, resources and research management?**<br>Research team, resources and research management?	An emminent team clear track record of success in this field and experts in the topic. However, it is a lot of professorial collaborators (many are psychologists and psychiatrists) and there is not too much detail on individual responsibilities and roles within the study. I also note an absence of mental health nursing and/or mental health social work from the co-apps. Given this is a study that includes an examination of usual practice in CMHTs as well as including a training package, it would seem valid to have representation from mental health nursing and/or social work researchers.
**Ethics and governance?**<br>Ethics and governance?	relapse into psychosis carries significant risk of harm to self and or others. The proposal only really focuses on the data security issues of the project. I note an absence of discussion about a risk register, reporting of adverse events and the management of an escalation of risk (even if this is to be managed by the case manager) there is also issues that may be raised during data collection and some assurances need to be made as to the training and support for the research assistant who will be collecting the data, including the need for a breach of confidentiality
**Patient and public involvement?**<br>Patient and public involvement?	This was not as thorough as it should be (or lacking in detail). I would have thought that given this is a self-management tool and promoting partnership working, I would have liked to have seen more service user and carer input into the actual proposal and study design itself. However, there are good plans to consult with service users and carers within WP1. I think not having qualitative data collection within the trial itself is a real limitation of this study and a missed opportunity to gather information on the processes which the outcome measures won't pick up.
**Research outputs?**<br>Research outputs?	There is a good plan for the dissemination of the project.

Question	Response
**What are the key strengths of this proposal?**<br>What are the key strengths of this proposal?	The key strengths is the use of mobile technology and real time data collection on early warning signs. It is a well designed feasibility study and has a team with a track record in this area of research.
**What are the key weaknesses of this proposal?**<br>What are the key weaknesses of this proposal?	lack of qualitative data collection during the actual trial which would greatly enhance the understanding of the experience of participating from both the clinician and service user and carer perspectives.<br>An absence of mental health nursing and/or social work within the research team<br>the absence of direct involvement of service users and carers within the development of the proposal itself.<br>lack of consideration of the variability of the case manager in the intervention, whether the whole team will all receive the training, how the service will be staffed during the training, and consideration that some of the case managers may not be present during the training and therefore not have been exposed to the intervention.<br>There is no consideration of excess NHS treatment costs involved in the intervention from the case manager perspective. Presumably if they have been trained to implement a stepped approach (in addition to TAU) then this time would constitute an NHS treatment cost.
**Questions for the applicants**<br>Do you have any questions for the applicants that you would like the opportunity for the applicants to respond to prior to the proposal being considered by the funding board?	1. how will the analysis take into account possible variance in case managers within the intervention arm.<br>2. How will you ensure that the case manager of the people recruited participates in the training?<br>3. please can you provide a rationale for whole team training when only 3-5 staff will be included in the study for recruitment of participants on their case loads.<br>4. can you justify why there isnt a mental health and/or social worker researcher as a co-applicant given that this will heavily rely on the work of these professional groups<br>5. Can you justify why you haven't included qualitative data collection during the trial itself (as well as fidelity measures)<br>6. what mental health risk training will be available for the peer supporter and research assistant and what plans do you have to produce a risk management

Question	Response
	protocol for the whole project given that relapse is a vulnerable time, and even discussing previous relapses may cause distress.<br>7. Can you explain why service users and carers have not been directly involved in the development of this proposal, and why you have opted for minimal involvement during the study (NB no service user co-apps, no PPI committee within the management structure) and will there be actual service users and carers in the steering committee?

Applicant Response To External Review

Applicant Response
